# Unintentional Ethylene Glycol Poisoning in an Adolescent

**DOI:** 10.7759/cureus.11521

**Published:** 2020-11-17

**Authors:** Rutul Patel, Anuja Mahesh Mistry, Chandrika M Mistry

**Affiliations:** 1 Internal Medicine, Smt. Nathiba Hargovandas Lakhmichand (NHL) Municipal Medical College (NHLMMC), Ahmedabad, IND; 2 Pathology, Saham Hospital, Saham, OMN

**Keywords:** ethylene glycol, oxalate crystals, fomepizole, ethanol, anion gap, car coolant, antifreeze

## Abstract

The differential diagnosis is broad when a patient presents with an altered mental status. Ethylene glycol poisoning, a life-threatening condition, can occur as an intentional self-harm attempt or unintentional consumption. It is metabolized in the liver by a series of enzymes, and the metabolites so formed are responsible for the majority of clinical effects. The diverse range of clinical effects includes central nervous system (CNS), gastrointestinal, cardiovascular system (CVS), pulmonary as well as renal effects. The evidence of metabolic acidosis, elevated anion gap, high osmolal gap, and calcium oxalate crystals in laboratory analysis strongly suggests ethylene glycol poisoning. The treatment traditionally consists of alcohol dehydrogenase inhibitors such as fomepizole or ethanol, and in some cases, hemodialysis is needed as well.

## Introduction

Ethylene glycol is a clear, colorless liquid at room temperature. It is commonly used in products such as automotive antifreeze, hydraulic brake fluids, and solvents. When used in automotive antifreeze, it often has a yellow-green fluorescent color [1]. Ethylene glycol is often ingested accidentally or intentionally, as it has a sweet taste. Ethylene glycol is absorbed rapidly through the gastrointestinal lining after ingestion and is subsequently metabolized in the liver by the enzyme alcohol dehydrogenase to glycolaldehyde and other toxic metabolites. Important laboratory tests in case of a known or suspected ethylene glycol poisoning are complete blood count (CBC), blood urea nitrogen (BUN), creatinine, lactate, serum electrolytes, ethylene glycol level, ethanol level, osmolarity, arterial blood gas (ABG) levels, and a urinalysis. Early intervention is crucial to prevent fatal complications such as renal failure. Antidotes, fomepizole, or ethanol should be administered intravenously as soon as possible to block the conversion of ethylene glycol to glyoxylic acid and prevent acidosis. Hemodialysis may be necessary in some cases to remove the parent compound as well as its toxic metabolites [2]. 

## Case presentation

An unconscious 13-year-old girl was brought to the emergency room (ER) at midnight by her parents. Symptoms started with nausea and abdominal pain in the evening, four hours before presentation to the ER. Her symptoms gradually progressed to vomiting, headache, and lethargy over the next two hours. The parents brought her to the hospital after she became unable to communicate and gradually lost her consciousness. Early physical examination revealed an unconscious patient with a Glasgow Coma Scale score of 5/15. Her vital signs showed tachycardia, tachypnea, and elevated blood pressure with a respiratory rate of 26/min, blood pressure 170/106 mmHg, and pulse 114/min. There was no associated pallor, jaundice, bleeding, lymphadenopathy, hepatosplenomegaly, cyanosis, or external physical injury signs. The detailed history given by parents did not reveal consumption of any unknown substances. 

The patient was immediately resuscitated with intravenous fluids while securing the airway and breathing. Random blood sugar testing revealed 84 g/dl, and other blood tests like complete blood count, liver function tests, lipid profile, C-reactive protein, creatinine, blood urea nitrogen (BUN), urinalysis and serum electrolyte were ordered. Serum electrolytes revealed concentrations of sodium 143 mmol/l; potassium 3.2 mmol/l; chloride 102 mmol/l; calcium 8 mg/dl; BUN 14 mg/dl; creatinine 1.02 mg/dl; urine examination revealed several calcium oxalate crystals with no pus/red blood cells/ albumin/ sugar. The computed tomography (CT) scan of the abdomen revealed no significant abnormality, and head CT was negative for stroke. There was no evidence of sepsis or post-ictal symptoms; glucose and beta-hydroxybutyrate were within normal limits.

The patient was then immediately shifted to the intensive care unit. Arterial blood gas analysis revealed the following: carbon-dioxide 44 mm of Hg, pH 7.1, and bicarbonate 10 mmol/L. These findings, along with an anion gap of 31 mmol/L, suggested a high anion gap metabolic acidosis. The osmolal gap was normal, with a value of 9 mOsm/L. We could not measure the blood level of ethylene glycol or ethanol due to the lack of this facility at the hospital. The presence of metabolic acidosis, elevated anion gap, calcium oxalate crystals on urine suggested ethylene glycol poisoning, but the lack of history of consumption of ethylene glycol did not support the diagnosis in question. Thus, from the circumstantial evidence and clinical presentation, it was presumed as a case of ethylene glycol poisoning, and empiric treatment was begun.

Although fomepizole is the standard FDA-approved treatment in this case, due to its unavailability, the patient was started on IV ethanol with a loading dose of 8 ml/kg of 10% solution, followed by a maintenance dose of 0.8 to 1.3 ml/kg/hour. Her condition slowly started to improve with the treatment. After regaining consciousness, she revealed that she mistook the green-colored car coolant as a soft-drink and consumed half a glass of it, approximately 80 ml. Laboratory investigations were repeated regularly to monitor the progress of the patient on the given treatment. On the third day, renal function tests revealed a high BUN value of 26 mg/dl and creatinine measuring 1.48 mg/dl, which necessitated hemodialysis initiation. The patient recovered fully and was discharged on the 10th day after three hemodialysis sessions in a span of seven days.

## Discussion

Ethylene glycol (EG) is commonly found in coolants, antifreeze solutions, solvents, and other household cleaners. Car coolant is a mixture of either ethylene or propylene glycol with water and is used in automobiles to overcome heating. It has a sweet taste due to the presence of ethylene glycol.

**Figure 1:**
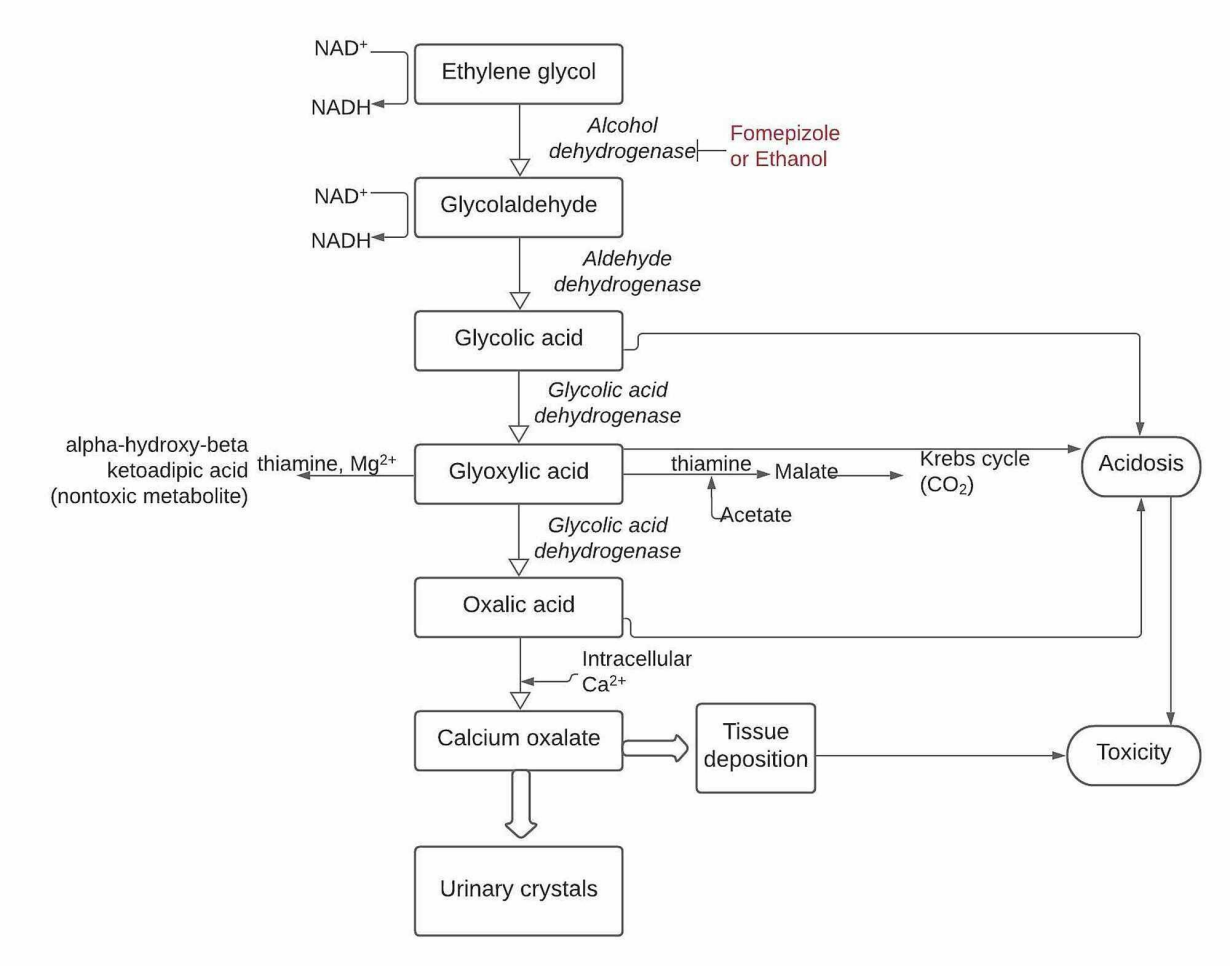
Metabolic pathways of ethylene glycol NAD - nicotinamide adenine dinucleotide; Mg^2+ ^- magnesium; Ca^2+^- calcium; CO_2 _- carbon dioxide

Ethylene glycol is rapidly absorbed from the gastrointestinal tract. It is metabolized in the liver first to glycolaldehyde by the action of alcohol dehydrogenase using nicotinamide adenine dinucleotide (NAD) in the process. Glycolaldehyde is further metabolized to glycolic acid, glyoxylic acid, and oxalic acid by the process of oxidation, as seen in Figure 1 [3-7]. Although ethylene glycol causes an increased osmolality and therefore an increased osmotic gap, it neither causes acidosis nor an increased anion gap by itself. The main cause of metabolic acidosis in ethylene glycol poisoning is the accumulation of its metabolites, particularly glycolic acid [8]. The oxalic acid can bind with calcium and precipitate to form calcium oxalate crystals. 

The clinical effects of ethylene glycol (EG) have been described to progress in three stages by Berman et al., which depend on the amount ingested and timing of intervention. The first stage is due to the central nervous system (CNS)-depressant effects that occur 30 minutes to 12 hours after ingestion and cause disinhibition, ataxia, slurred speech, and incoordination. Severe poisoning can also alter consciousness with progression to coma, associated with seizures, hypotonia, hyporeflexia, meningism, and absent pupillary reflexes [9]. Gastrointestinal effects include nausea, vomiting, hematemesis, abdominal pain, and cramping. Some autopsy findings also show calcium oxalate deposits in intestinal mucosa and focal hemorrhages in the gastric lining and liver [2]. Cardiopulmonary effects constitute the second stage that occurs 12-48 hours post-ingestion, leading to secondary pulmonary edema, which could be due to cardiac failure, adult respiratory distress syndrome, or aspiration of gastric contents [10]. The renal effects mark the final stage, occurring after 48 hours since ingestion, and is caused by the deposition of calcium oxalate crystals within the tubules. Clinically, it may present with flank tenderness, proteinuria, elevated creatinine, eventually leading to anuria [9]. Other systemic effects include tetany/myoclonus resulting from hypocalcemia (as calcium is consumed for the formation of calcium oxalate crystals), myalgia, myositis, diffuse internal hemorrhage in the brain, lungs, and heart [2].

The diagnosis of ethylene glycol toxicity is made by combining the history of EG consumption, clinical symptoms, and laboratory studies. In the absence of known consumption history, diagnosis relies on identifying high anion gap metabolic acidosis, blood ethylene glycol level, and calcium oxalate crystals in urine. The measurement of serum EG levels by gas chromatography has also been employed to confirm the diagnosis [11]. 

The anion gap that measures the concentration of acids in the blood is calculated as follows: [Na^+ ^- (HCO_3_^- ^+ Cl^-^)]. A normal anion gap is usually between 8 to 12 mEq/L. The anion gap measurement is critical for diagnosing any toxic alcohol poisoning, but in the case of EG toxicity, the level may not be elevated for several hours after ingestion since EG itself is not an acid. Once it starts to metabolize into toxic compounds (which are acidic), the anion gap metabolic acidosis becomes evident. An uninhibited alcohol dehydrogenase enzyme has a half-life of three to eight hours, which increases to more than 17 hours after the administration of ethanol/fomepizole. Therefore, at least three hours after ingestion are required to pass for the formation of its acidic metabolites [12].

Ethylene glycol is an osmotically active substance, and thus calculating an osmolal gap [measured osmolality - {2[Na^+^] + glucose/18 + BUN/2.8 + ethanol/4.6}] is another screening test that can narrow the differential diagnosis in cases of anion gap metabolic acidosis. A normal osmolal gap is usually less than 10 mOsm/L. Osmolal gap will be higher during the early hours after ingestion as the concentration of EG is higher. Gradually as the toxic acids are generated from the oxidation of ethylene glycol, the osmolal gap eventually returns to normal [12]. 

**Figure 2:**
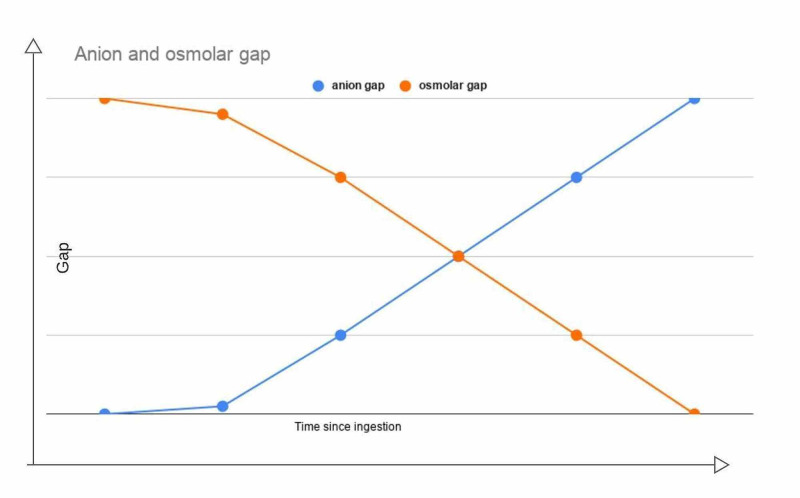
Two different peaks of anion gap and osmolal gap relative to the time of ingestion

It needs to be emphasized that the osmolal gap and anion gap will show a peak at two different extremes during the time course of poisoning. An elevated osmolal gap and no acidosis are expected during the early period after poisoning; a high anion gap and no osmolal gap are expected in the later period, as shown in Figure 2 [12].

Several differential diagnoses need to be considered in case of anion gap metabolic acidosis, including toxicity with methanol, salicylates, paraldehyde, iron, isoniazid; non-toxic causes constitute diabetic ketoacidosis (DKA), uremia, and lactic acidosis. DKA being the most common cause of metabolic acidosis in adolescents, needs to be ruled out first. Metabolic acidosis with increased anion and osmolal gaps are seen in methanol and ethylene glycol toxicity, but more common manifestations of methanol poisoning include visual disturbances and papilledema due to the production of formic acid.

Treatment guidelines: considering the various life-threatening acute complications of ethylene glycol poisoning, such as acid-base metabolic disturbances, acute renal failure, and CNS depression, stabilizing the patient’s condition takes the highest priority by securing the patient’s airway, breathing, and circulation [4].

Gastric decontamination: the next priority is to eliminate as much ethylene glycol from the gastrointestinal tract as possible (in case of ingested toxicity), with gastric aspiration or lavage proven to be most effective if administered within an hour of ingestion due to its rapid absorption rate. Intravenous (IV) 5% dextrose should be administered empirically for suspected hypoglycemia, and IV short-acting benzodiazepines or IV phenytoin is recommended for the treatment of seizures. The associated hypocalcemia is not generally treated with IV calcium gluconate unless severe because the infused calcium may act as a catalyst for further formation of calcium oxalate crystals [4].

Fomepizole and ethanol: act by competitively inhibiting the enzyme, alcohol dehydrogenase. This enzyme has a much greater affinity for ethanol and fomepizole than ethylene glycol, which preferentially catalyzes the metabolism of ethanol, thus halting the metabolic pathways of ethylene glycol and preventing its further absorption and breakdown into active metabolites. Furthermore, the unabsorbed ethylene glycol becomes a target for gradual urinary elimination [5].

The indications for fomepizole/ethanol for the management of EG poisoning include [5,6] serum ethylene glycol level >20 mg/dL OR documented history of recent ingestion of toxic amounts of ethylene glycol in conjunction with a serum osmole gap >10 mOsmol/L OR history or strong clinical suspicion of ethylene glycol poisoning in conjunction with at least two of the following criteria: arterial blood pH <7.3, plasma bicarbonate or serum CO2 content <20 mmol/L, serum osmole gap >10 mOsmol/L, calcium oxalate crystalluria.

Dosage recommendations for fomepizole in EG poisoning for patients not undergoing hemodialysis is a loading dose of 15 mg per kilogram of body weight, followed by 10 mg per kilogram every 12 hours; after 48 hours, 15 mg per kilogram every 12 hours. For patients undergoing hemodialysis, the same doses administered to patients who are not undergoing hemodialysis, except that the drug is given six hours after the first dose and every four hours thereafter [3]

Dose recommendations for ethanol in EG poisoning: during ethanol infusions, the goal is to maintain a serum ethanol concentration between 80 to 120 mg/dl, which is the optimum dose at which it effectively inhibits alcohol dehydrogenase [13]. The treatment requires a single loading dose and subsequent maintenance doses depending on EG's volume ingested the renal function and ongoing doses for patients on dialysis. The loading dose required can be calculated as a multiplication product of the goal plasma concentration of ethanol (example, 100 mg/dl), its volume of distribution (0.6L/kg), and the patient’s weight. However, empiric ethanol administration as a 10% solution with a loading dose of 8 mL/kg in 30-60 minutes, followed by maintenance doses of 1-2 mL/kg/hour, has proven to be potentially effective [14].

Among the two, only fomepizole has been approved by the US Food and Drug Administration for the treatment of EG poisoning [4]. Although conventionally used, ethanol also poses various side effects that limit its utilization. These effects include but are not limited to, the following: potential for hypoglycemia, inebriation, sedation, the potential for volume overload, and the need for continuous laboratory monitoring [5]. 

Hemodialysis: while an ethanol infusion can prevent most of the metabolism of ethylene glycol, the parent compound, aldehyde metabolites, and organic acids must also be removed [15]. The most efficient way to remove the compound is by hemodialysis. Hence, the critical importance of hemodialysis in the treatment must be emphasized, especially in patients where a significant amount of time has elapsed between EG ingestion and therapeutic intervention with ethanol/fomepizole; or in cases where the history is either unreliable or unobtainable, such as in this case of an unconscious patient. The current guidelines for initiation of hemodialysis include ^A^: 1) worsening clinical status; 2) significant metabolic acidosis (arterial blood pH <7.3); 3) renal failure; 4) visual disturbance; 5) electrolyte abnormalities; 6) serum ethylene glycol level >50 mg/dL ^B ^(^A^Any of the listed criteria is considered indications if unresponsive to standard measures and supportive treatment; ^B ^This is a conventional indication, but recent evidence and guidelines suggest that hemodialysis may not be necessary in some cases if this is the only criterion that is satisfied, the patient is asymptomatic, and the arterial pH is normal [5]).

The required time for dialysis can be calculated by a validated formula [16]: [-V *ln* (5/A)/0.06*k*], where V = total body water (TBW) in liters estimated by the Watson’s formula [17] (*ln - *natural logarithm; A - initial toxin concentration in mmol/L; *k* is the 80% of the manufacturer-specified dialyzer urea clearance in milliliters per minute at the initial observed blood flow rate). Being highly dialyzable, fomepizole, and ethanol require more frequent dosing for patients on dialysis.

In the clinical scenario, a rebound increase in the plasma EG concentration has been noticed within 12-36 hours after discontinuing hemodialysis, attributable to its redistribution from tissue to the plasma compartment. This necessitates the continuous use of fomepizole or ethanol with ongoing monitoring of anion gap, osmolar gap, electrolytes, and serum osmolarity. Fomepizole/ethanol can be discontinued if the following criteria are met: serum EG becomes either < 20mg/dl or undetectable, normalization of anion gap/pH with the absence of any symptoms. 

Thiamine and magnesium: although it is commonly employed in patients with chronic alcohol abuse, it has been theoretically proven to have some therapeutic importance in cases of acute ethylene glycol poisoning due to the minor role it plays in its metabolic pathway. In the presence of thiamine and magnesium, the glyoxylic acid is converted to a non-toxic compound, alpha-hydroxy-beta-ketoadipic acid. Therefore, empiric IV thiamine and magnesium are hypothesized to be of some clinical benefit for thiamine deficient patients with alcohol toxicity [4].

Sodium bicarbonate: the conventional effectiveness of sodium bicarbonate in managing various forms of metabolic acidosis forms the rationale for its use in ethylene glycol poisoning to correct pH. Although the extreme acidemia in EG poisoning may not be absolutely correctable with boluses of NaHCO_3_, nevertheless, it has been employed for patients with arterial pH less than 7.3 [5].

## Conclusions

It can often be challenging to diagnose and manage an unconscious patient, especially in this age group, due to the wide range of possible differentials. Several etiologies are considered in this patient population with high anion gap metabolic acidosis, the most common being diabetic ketoacidosis and salicylate toxicity. This study emphasizes the crucial role of laboratory testing in support of the diagnosis. Given the unavailability of fomepizole, the use of ethanol, as well as hemodialysis, can be life-saving in patients who present later during the time course of poisoning. Considering the unsupervised attempt by the patient to consume ethylene glycol and the unclear circumstances surrounding the patient, we felt the necessity to consult her for a psychiatric evaluation. The patient was followed up for a period of four weeks that suggested an absence of any psychiatric illness. To prevent such future episodes, the parents were counseled on the contents and safety profile of car coolants and other similar substances and were advised to keep these products out of children's reach. 
